# Radiation-quality-dependent bystander effects induced by the microbeams with different radiation sources

**DOI:** 10.1093/jrr/rrt219

**Published:** 2014-03

**Authors:** M. Suzuki, N. Autsavapromporn, N. Usami, T. Funayama, I. Plante, Y. Yokota, Mutou, M. Suzuki, H. Ikeda, Y. Hattori, K. Kobayashi, Y. Kobayashi, T. Murakami

**Affiliations:** 1 National Institute of Radiological Sciences, Chiba, Japan; 2 High Energy Accelerator Research Organization, Tsukuba, Japan; 3 Japan Atomic Energy Agency, Takasaki, Japan; 4 NASA Johnson Space Center, Houston, USA

**Keywords:** Bystander effect, Microbeam, Gap junction, P53, HPRT

## Abstract

A central paradigm in radiation biology has been that only cells ‘hit’ by a track of radiation would be affected to induce radiobiological consequences, and cells ‘not hit’ should not be. This is the basis of the current system for risk estimation of radiobiological effects. However, it has recently been challenged by so-called non-targeted effects, such as bystander effect, and such radiation-induced cellular responses may have important implications for risk evaluation of low-dose-rate radiations as well as in tumor radiotherapy. Our group has been studying radiation-quality bystander cellular effects using the microbeams with different radiation sources.

It is essentially important for evaluating risk such a low-dose-rate exposure as the accident of Fukushima Daiichi Nuclear Power Plants to examine bystander effects induced by low-LET electromagnetic radiations, such as X or gamma rays. We have been studying the cellular responses in normal human fibroblasts by targeted cell nucleus irradiations with monochromatic X-ray microbeams (5.35 keV) produced by Photon Factory in High Energy Accelerator Research Organization. The results indicated that the bystander effect in cell- killing effect was observed in the targeted cell nucleus irradiation, not in the random irradiation containing both cell nucleus and cytoplasm by Poisson distribution. The results suggest that energy deposition in cytoplasm is an important role of inducing bystander effects in case of low-LET radiations.

We have also been investigating high-LET-radiation induced bystander effects using the heavy-ion microbeams at Takasaki Ion Accelerators for Advanced Radiation Application in Japan Atomic Energy Agency. Only 0.04% of the total numbers of normal human fibroblasts were irradiated with C-ion (220 MeV), Ne-ion (260 MeV) and Ar-ion (460 MeV) microbeams collimated at 20 μm in diameter. Cell-killing effect and gene mutation at HPRT locus in the cells irradiated with C ions were higher beyond our expectations and returned the estimated values that only 0.04% of the total cells were irradiated when using the specific inhibitor of gap junctions. On the other hand, no induced biological effects were observed in Ne and Ar ions whether the inhibitor was applied or not. The result suggested that the C-ion microbeam was capable of inducing bystander cellular effects via gap junction-mediated cell-cell communication. There is clear evidence that bystander cellular effects are dependent on radiation quality.

It is also important for highly developed heavy-ion radiotherapy to identify bystander effects induced by spatially low-fluence irradiations with heavy-ion beams. We have been investigating the biological effects using human tumor cell lines. The results clearly showed that bystander effects were observed in the carbon-ion irradiation but not in other ions as well as the effects in normal fibroblasts. Furthermore, the bystander cell-killing effect in tumor cell lines was strongly induced in the cells harboring wild-type P53 not in mutated-type P53 cells. The results provide the important implication for a tailor-made therapy using carbon ions.

